# Swine influenza virus surveillance programme pilot to assess the risk for animal and public health, the Netherlands, 2022 to 2023

**DOI:** 10.2807/1560-7917.ES.2025.30.22.2400664

**Published:** 2025-06-05

**Authors:** Erhard van der Vries, Evelien A Germeraad, Annelies Kroneman, Lucía Dieste-Pérez, Dirk Eggink, Eveline Willems, Alexander MP Byrne, Nicola Lewis, Manon Houben, Ron Fouchier, Adam Meijer, Joke van der Giessen

**Affiliations:** 1 Royal GD, Deventer, the Netherlands; 2 Wageningen Bioveterinary Research (WBVR), Lelystad, the Netherlands; 3 National Institute for Public Health and the Environment (RIVM), Center for Infectious disease Control, Bilthoven, the Netherlands; 4 Worldwide Influenza Centre (WIC), World Health Organization (WHO) Collaborating Centre for Reference and Research on Influenza, The Francis Crick Institute, London, United Kingdom; 5 Department of Viroscience, Erasmus University Medical Center (MC), Rotterdam, the Netherlands.

**Keywords:** Influenza, swine influenza one health epidemiology, One Health, H1N1, H3N2, phylogenetics

## Abstract

**Background:**

Swine influenza has a considerable impact on pig populations and poses a pandemic threat to humans. However, little is known about the influenza A viruses circulating among pigs in the Netherlands.

**Aim:**

We piloted a surveillance programme aimed at enabling swine influenza A virus (swIAV) surveillance in the Netherlands: investigated prevalence, genomic characteristics and recent evolution of circulating swIAV variants and compared them with relevant human and swine influenza viruses from the Netherlands and other European countries.

**Methods:**

We collected and tested respiratory samples from pigs (2019–2023) for swIAV, characterised the viruses with molecular and virological methods and shared molecular data of swine and relevant human influenza A viruses in a national platform.

**Results:**

We detected swIAV throughout the year in 342 (42%) of 824 respiratory samples from 90 farms. Complete genome sequencing identified 73 H1N1, 51 H1N2 and one H3N2 viruses. Phylogenetic analyses identified viruses from each of the three H1 swine lineages (1A/B/C) and four subclades. Viruses from the 1A lineage clustered into three subgroups with distinct antigenic properties, which seemed descendent from separate introductions of human seasonal A(H1N1)pdm09 viruses. Phenotypically, no reduced susceptibility to existing antiviral drugs oseltamivir and zanamivir was found.

**Conclusion:**

We provided insights into swIAVs in pigs in the Netherlands, including antiviral susceptibility and antigenic differences. It highlighted occasional virus transmission between humans and pigs. Sharing swIAV data at a national level will be continued to reduce influenza burden in swine and support identification and characterisation of emerging swIAVs with zoonotic potential.

**Key public health message**

**What did you want to address in this study and why?**

Swine influenza viruses cause disease in pigs but sometimes in humans too. We wanted to study how common these viruses are in pigs in the Netherlands and learn more about them. We collected genetic information of these viruses and created a national online data platform to compare and analyse genomes of swine and human influenza viruses for trend and risk analyses for public health.

**What have we learnt from this study?**

Swine influenza viruses are found throughout the year on Dutch pig farms. Viruses descending from the 2009 influenza pandemic outbreak spread repeatedly from humans to pigs afterwards. These viruses are genetically similar, sensitive to all existing antiviral drugs, but different in the way they are recognised by serum antibodies. This difference may affect diagnostic detection and effectiveness of swine influenza vaccines.

**What are the implications of your findings for public health?**

We established a network and gathered more information about the distribution, origin and characteristics of swine influenza viruses in the Netherlands. With this information and the established network, we can react faster to infections that spread between pigs and humans in the future.

## Introduction

Swine influenza A viruses (swIAV) are enzootic in pigs in Europe and cause respiratory disease in pigs [1]. Clinical signs include fever, coughing, nasal discharge, sneezing, laboured breathing and loss of appetite. As pigs are also susceptible to influenza A viruses from birds and humans, they can facilitate the occurrence of co-infections from which influenza viruses may arise with reassorted genomes [2,3]. These new viruses pose considerable risks if they acquire the ability to cross species barriers and transmit efficiently between humans. However, and in contrast to the data available on viruses in humans, little is known about the circulation of influenza A viruses in pigs [1]. The threat posed by circulating swIAV is increasingly recognised by European and other international health organisations and policymakers as a zoonotic risk after the 2009 influenza A(H1N1) and COVID-19 pandemics [3-5]. Recent events underline the need to better characterise the circulating influenza A viruses in pigs. First, Eurasian avian-like H1N1 G4 viruses with increased zoonotic potential were detected in pigs in China in 2020 [6]. Next, in 2022 and 2023, swIAV infections in humans were reported in European countries, including three patients in the Netherlands and one patient in the United Kingdom (UK) [2,7]. Finally, ongoing outbreaks of highly pathogenic avian influenza (HPAI) H5N1 (clade 2.3.4.4b) in farm animals, including poultry [8], fur animals [9], cattle [10], backyard pigs and recent zoonotic infections in humans in the United States (US), show that similar events in pigs do not seem unlikely, and justify the call for increased influenza A virus surveillance in these animals [11]. The relevance for such surveillance at a national level became clear in 2022 after a Dutch zoonotic H1N2v infection with a swine virus from the 1C Eurasian avian lineage [7]. At that time, relevant questions on cross-species transmission and assessment of public health risks could be addressed through close collaboration between public health and animal health institutions.

To gain insight into the occurrence, potential risk, genomic characteristics and recent evolution of swIAV, a national pilot project was set up in pigs in 2022. The aim of this study with a One Health (OH) focus was to characterise swIAV circulating in the Netherlands and to compare those with relevant human and swine influenza viruses from the Netherlands and other European countries, for instance, with those collected by the European Swine Influenza Network (ESFLU) and World Health Organization (WHO) Global Influenza Surveillance and Response System. Collaboration between animal health and public health professionals is essential in OH surveillance. Therefore, this pilot project was built on collaboration between the National Institute of Public Health (RIVM), Royal GD, Erasmus Medical Center (EMC) and Wageningen Bioveterinary Research (WBVR) using a joint online platform.

## Methods

### Sample collection

In 2022, the Dutch pig industry counted 11.3 million pigs and 3,272 pig farms. Samples from pigs were collected via two collection routes.

Route 1 included samples collected between 1 June 2022 and 7 July 2023 from pigs with influenza-like clinical signs. Veterinarians of Dutch pig farms were approached to participate in this pilot project on a voluntary basis. In total, 30 veterinarians from 10 veterinary practices responded covering all three regions in the Netherlands. The veterinarians were asked to identify farms with pigs showing influenza-like clinical signs and collect five individual nose swabs (FLOQ Swabs, Copan, Italy) from affected pigs and group saliva (oral fluid) samples (Happy bite sampling kit, VDGEB086, Royal GD, Deventer, the Netherlands) from four affected groups. The total number of pigs per group varied and was not recorded. There were no selection criteria for farm type, so both slaughter pig farms and farrowing farms were included. Epidemiological data on presence and characteristics of clinical signs, age group of the affected pigs, farm location, outdoor access of pigs and presence of other animals on the farm were also collected.

The collection Route 2 provided extra samples sent in for routine veterinary diagnostics to Royal GD between 2019 and 2023. These samples were included to further increase the number of total virus genome sequences. This collection consisted of respiratory samples (nasal and trachea-bronchial swabs) and post-mortem lung tissues with an influenza-positive PCR test from pigs submitted for necropsy but not necessarily with a suspected respiratory virus disease.

All metadata, including sample identification codes (ID), were pseudonymised before submission to the database. Farm location was assigned to one of the three regions (northern, central, southern) in the Netherlands.

### Virological investigations

Nasal swabs and saliva samples collected specifically for the pilot project were sent to Royal GD and aliquoted upon arrival. A backup sample was stored at < −60°C. The samples were tested for the presence of the influenza A virus matrix gene segment by RT-PCR [12]. Influenza H1/H3 and N1/N2 subtyping was done for those viruses of which no haemagglutinin (HA) and/or neuraminidase (NA) sequence was obtained using a commercial VetMAX-Gold SIV subtyping PCR kit (Thermo Fisher, Waltham, US). Virus isolation was attempted on PCR-positive samples with quantification cycle (Cq) values < 32 by culturing these samples in Madin-Darby canine kidney (MDCK)- 2,6-sialyltransferase (SIAT)/MDCK-I mixed cell cultures.

### Complete genome sequencing

Whole genome sequencing (WGS) was performed at Royal GD and WBVR using RNA from the original material (Cq < 32) and virus isolates using two different approaches. At Royal GD, WGS was performed using Nanopore sequencing as described previously [13]. For this approach, viral RNA was isolated using the MagMAX Pathogen RNA/DNA kit (Applied biosystems, Thermo Fisher) and amplified using a universal eight-segment PCR. The PCR amplicons were purified using AMPure XP magnetic beads (Thermo Fisher) and further processed using the native barcoding and ligation sequencing kits (Oxford Nanopore, Oxford, UK) followed by sequencing on a GridION Oxford Nanopore platform with R9.4.1 flow cells. Base calling and demultiplexing was done using Guppy version 6.3.8 (https://nanoporetech.com/software/other/guppy) with super-accurate base calling. FastQC files contained QC-passed reads with Cq value cutoff > 10. Finally, consensus sequences were obtained using the ViroConstrictor pipeline [14]. At WBVR, Illumina sequencing was performed with RNA extracted at Royal GD, as described previously [15]. The consensus sequences were determined in the CLC Genomics Workbench (QIAGEN, Hilden, Germany) using a reference set of avian influenza viruses supplemented with reference sequences of swIAV.

### Molecular data management platform

A database was set up within the molecular data management platform (MPF). This online platform is a secure online environment previously created by RIVM. It is a tool to share sequences among the consortium partners and perform phylogenetic analyses and visualisations. All swIAV sequences and metadata generated were uploaded to the MPF. The platform was supplemented with a reference set of publicly available swine and human influenza viruses. At the time of the analysis, this set consisted of 120 H1Nx and 19 H3N2 swine viruses from different countries, 193 human H1N1pdm09 (2014–2023) and 319 human H3N2 (2014–2023) viruses representative of genomic diversity in the Netherlands, and 10 viruses from human zoonotic infections with a swine virus (1986–2023) from the Netherlands. The sequences of 24 WHO human H1N1pdm09 and H3N2 vaccine candidates (2011–2023) were also added, as well as the viruses of the Ceva Respiporc Flu3 and FluPan porcine vaccine viruses (https://swine.ceva.com). All new swine influenza sequences analysed within the MPF environment were uploaded to GISAID (https://gisaid.org). For reference, a table with names of all virus isolates, including GISAID ID, subtype and HA clade assignments is listed in Supplementary Material. Phylogenetic analyses of the H3N2 viruses were performed at the Worldwide Influenza Centre (WIC) using a set of swine H3 sequences (2009–2024) downloaded from GISAID.

### Phylogenetic analyses

Lineage assignment of the internal segments was performed using a selection of the reference sequences with clades assigned using octoFLU [16]. Clade assignment was performed using the bv-brc subspecies classification tool with the Orthomyxoviridae – Swine influenza H1 reference set [17]. Tree building and visualisation was performed using UFBoot2 [18], ModelFinder [19], MAFFT [20], IQtree [21] and TreeViewer [22].

### Antiviral testing

For all virus isolates and a subset of the non-cultured original specimens with high virus load, amino acid sequences of the M2 protein (M2), NA and polymerase acidic protein (PA) were analysed for markers indicative of reduced susceptibility of human seasonal influenza A viruses to adamantane drugs (M2-blockers), NA inhibitors and baloxavir marboxil, respectively. Sequence analysis for drug reduced susceptibility markers was done manually using amino acid alignments in BioEdit version 7.2.5 (https://bioedit.software.informer.com/7.2) and FluSurver [23]. In total, 36 virus isolates, 17 H1N1 and 19 H1N2 viruses were also tested phenotypically for susceptibility to oseltamivir and zanamivir using an in-house MUNANA-based assay [24].

### Antigenic characterisation

A representative subset of virus isolates was selected based on the genetic differences in the HA segment. A total of 36 virus isolates were tested in the hemagglutination inhibition (HI) assay using turkey erythrocytes and ferret antisera for antigenic characterisation as previously described [25]. For this purpose, new ferret antisera were raised against viruses from the 1A classical lineage clade 1A.3.3.2 (A/Swine/Netherlands/719/19 and A/Swine/Netherlands/4322/20), clade 1B.1.2.1 (A/Swine/Netherlands/8836/22 and A/Swine/Netherlands/5591/22), 1C.2.1 (A/Swine/Netherlands/11135/22) and 1C.2.2 (A/Swine/Netherlands/6151/23), because there was no or low reactivity of the viruses with the available sera. Ferret antisera raised against a virus from the 1C Eurasian avian clades 1C.2.1 (A/Swine/Netherlands/3315/16), 1C.2.2 (A/Hessen/47/20), 1C.2.3 (A/Hebei-haigang/1572/19), and human seasonal influenza virus (A/California/07/09) were obtained from the WIC. In total, we used twelve ferret antisera, raised against three clade 1A.3.3.2, two clade 1B.1.2.1 and seven clade 1C viruses of pigs and humans (zoonoses).

## Results

### Demographics and pig farm characteristics

**Figure 1:**
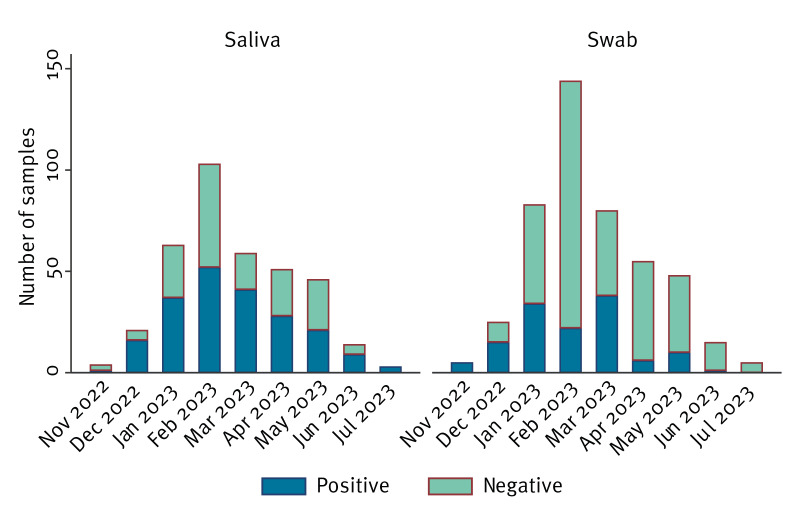
Survey of influenza in pigs on pig farms, by sample type and month, the Netherlands, November 2022–July 2023 (n = 90)

In this pilot project, nasal and saliva swabs from pigs on 90 farms were collected (Route 1). The farms were distributed across the country and farm locations were allocated to the northern (24/90; 27%), central (8/90; 9%) and southern (49/90; 54%) regions of the Netherlands. The latter region is the area with the highest pig density [26]. Most farms were exclusively pig production farms, and pigs were kept indoors on 87 (97%) farms. However, three pig farms (3%) had outdoor facilities, and 13 (14%) farms kept animals beside pigs, including pig farms with poultry (4/90; 4%), ruminants (7/90; 8%) and horses (2/90; 2%). However, the farm location was not provided in all cases (9/90; 10%). Also, 11 farms (12%) did not provide information about housing of other animals. Influenza A virus was detected on 78% (70/90) of farms by PCR throughout the entire study period (Figure 1).

### Collecting and processing of pig samples

In total, 824 respiratory samples were collected from pigs showing influenza-like clinical signs to increase the likelihood of obtaining a virus-positive sample (Route 1; Table 1). Signs included fever, lethargy and respiratory signs (e.g. nasal discharge, sneezing, coughing). Samples consisted of either saliva from groups of pigs (n = 364) or nasal swabs from individual animals (n = 460) with the highest proportion of influenza-positive samples from saliva (211/ 364; 58%) as compared with individual nasal swabs (131/460; 28%). Most samples were collected from January to May, but virus was detected year-round. To obtain a representative set of influenza virus isolates for phenotypic antiviral susceptibility testing and antigenic characterisation, virus isolation was attempted by incubating a subset of samples with sufficient virus loads (quantification cycle (Cq) < 32) in MDCK-SIAT/MDCK-I cell culture.

**Table 1:** Detection of influenza virus in respiratory specimens in a survey of influenza in pigs, the Netherlands, 2019–2023 (n = 900)

Sample type	Collection route	Number	PCR result			
			Positive	%	Negative	%
Saliva (group)	1	364	211	58	153	42
Nasal swab (individual)	1	460	131	28	329	72
Lung	2	46	46	100	0	0
Other respiratory sample	2	30	30	100	0	0

### Complete genome sequencing analysis of swine influenza viruses

Of 342 (342/824; 42%) influenza-positive samples from Route 1, 125 (in)complete influenza A virus genomes were obtained. Subtype assignment (by sequencing and PCR) revealed all viruses were of the H1N1 (73/125) or H1N2 (51/125) subtypes, except for a single H3N2 subtype detection (1/125) (Table 2). An additional 46 influenza-positive lung tissues and 30 respiratory samples, collected during routine diagnostics at Royal GD, were added at this stage to increase the total number of virus sequences and isolates (Route 2; n = 35).

**Table 2:** Characterisation of swine influenza viruses in a survey of influenza on pigs, by subtype and haemagglutinin clade, the Netherlands, 2019–2023 (n = 125)

Subtype	Total	H1 clade				
		1A.3.3.2	1B.1.2.1	1C.2.1	1C.2.2	Unassigned
H1						
H1N1	73	2	2	7	55	7
H1N2	51	23	16	2	9	1
H3 (Clade 2000.3)						
H3N2	1	Not applicable				0

H: haemagglutinin; N: neuraminidase.

### Human and swine influenza viruses

**Figure 2:**
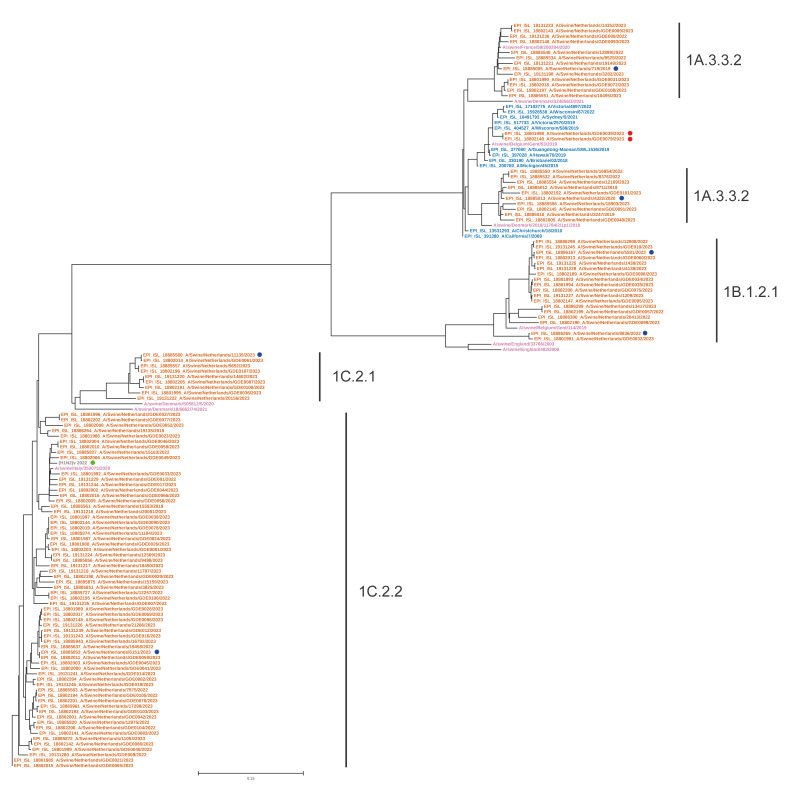
Phylogenetic tree for the haemagglutinin of swine influenza H1 viruses, Europe, 2009–2023 (n = 109)

Two swine influenza A subtypes (H1N1 and H1N2) circulated on pig farms in the Netherlands between 2019 and 2022. The circulating swine H1 viruses in pigs included those from the 1A classical lineage, the 1B seasonal lineage associated with the 1990s human-to-swine transmission and the 1C Eurasian avian lineage (Figure 2) [27]. Virus sequences in the H1 phylogenetic tree clustered into clades 1A.3.3.2, 1B.1.2.1, 1C.2.1 and 1C.2.2 (Figure 2). The swine H1 viruses clustering in the 1A.3.3.2 clade with the human vaccine strains (2011–2023) seem to have been introduced from humans in at least three different occasions since 2009. For example, two identical viruses collected on a single farm clustered with human H1N1pdm09 viruses from the 2019–2020 influenza season (HA clade 6B.1A.5a.2a) albeit with long branch lengths from the putative human seasonal ancestor due to under-sampling in pigs (Figure 2, red dot). Conversely, during this pilot project, an H1N2v infection originating from pigs was identified in a human patient in the Netherlands, following unprotected contact with pigs 3 weeks before the hospital visit. The patient had received a stem cell transplantation 1.5 years before the infection. This virus was genetically related to the viruses isolated in this project (Figure 2, green dot). For more information about swIAV infections in humans detected 2020–2023 in the Netherlands, including the H1N2v one, see the paper by Eggink et al. [7].

HA: haemagglutinin.

HA of the viruses were from three lineages (1A-C) and four HA clades (1A.3.3.2; 1B.1.2.1; 1C.2.1; 1C.2.2). Orange: viruses from pigs.; blue: human reference vaccine strains (2009–2022); pink: swine reference viruses from other European countries (e.g. Belgium, Denmark, Italy, France, the United Kingdom).

Red dots mark an example of a recent anthroponotic event. Blue dots mark viruses selected for the generation of ferret reference sera for HI testing. Reference viruses A/Swine/Netherlands/719/19 and A/Swine/Netherlands/4322/20 represent two antigenically distinct subgroups within the 1A.3.3.2 clade. Green dot marks an H1N2v isolated from a Dutch patient.

Only one H3N2 virus was detected during the study period. This virus was detected in February 2023 in the central region (GISAID accession number ID: EPI_ISL_18802007). The BV-BRC H3 classification tool revealed that the HA and NA gene segments clustered with swine H3N2 viruses circulating in Germany, Denmark and the Netherlands between 2013 and 2016, although only limited genetic information is available in most European countries. The H3 sequence clustered with those from the 2000.3 clade, the closest virus sequence being A/swine/Germany/AR10832/2016. The internal genes appeared to be derived from the 1A classical lineage, like other swine H3N2 viruses circulating in this period in Europe but did not appear to be circulating currently (data available on request). Unfortunately, isolation of this H3N2 virus was not successful. Of the 125 H1 viruses, 109 could be fully sequenced. Lineage assignment of the six internal segments segregated them into groups with internal segments of the 1A classical swine (CS) or 1C Eurasian avian (EA) lineage (Table 3). Those from the second group only shared the M gene segment from this group complemented with five segments belonging to the 1C EA virus lineage. These two internal lineage patterns were found for the H1 subtypes from the 1A.3.3.2 as well as the 1C.2.1 lineages. Viruses from the 1B seasonal (HS) lineage all had the second pattern.

**Table 3:** Characterisation of swine influenza viruses in a survey of influenza in pigs, by internal gene composition, the Netherlands, 2019–2023 (n = 109)

HA		NA	Internal gene segments						Sequences	
Lineage	Clade	Subtype	PB2	PB1	PA	NP	M1	NS	n	%
1A	1A.3.3.2	N1	CS	CS	CS	CS	CS	CS	2	2
1A	1A.3.3.2	N2	CS	CS	CS	CS	CS	CS	15	14
1A	1A.3.3.2	N2	EA	EA	EA	EA	CS	EA	6	6
1B	1B.1.2.1	N1	EA	EA	EA	EA	CS	EA	2	2
1B	1B.1.2.1	N2	EA	EA	EA	EA	CS	EA	16	15
1C	1C.2.1	N1	CS	CS	CS	CS	CS	CS	4	4
1C	1C.2.1	N1	EA	EA	EA	EA	EA	EA	3	3
1C	1C.2.1	N2	EA	EA	EA	EA	EA	EA	1	1
1C	1C.2.2	N1	EA	EA	EA	EA	EA	EA	52	48
1C	1C.2.2	N2	EA	EA	EA	EA	EA	EA	8	7

CS: classical swine virus; EA: Eurasian avian; HA: haemagglutinin; HS: human seasonal virus; NA: neuraminidase.

At farm level, more than one virus was sequenced from 21 farms (21/ 90; 23%). In most cases, the sequenced viruses were highly similar, but in four (4%) cases, sequencing yielded viruses that were genetically distinct. In three of four cases, these viruses differed from each other for all segments, which indicates simultaneous co-circulation of different H1N1 variants on a single farm.

### Antiviral susceptibility testing

**Figure 3:**
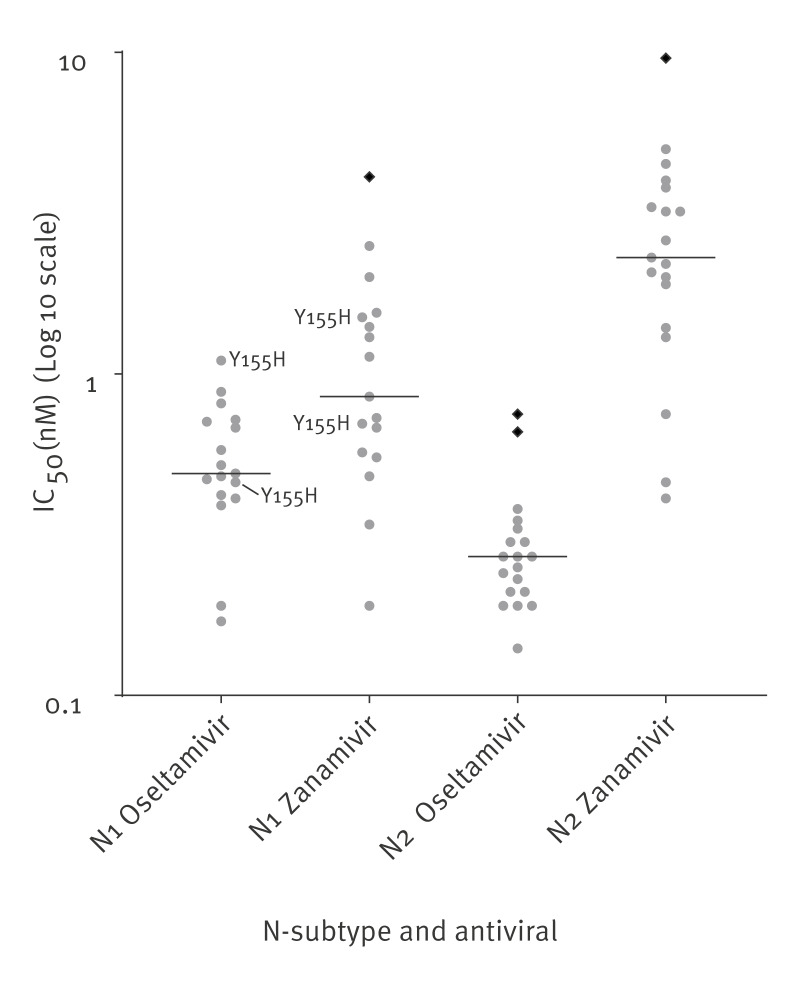
Half maximal inhibitory concentration (IC_50_) values for oseltamivir and zanamivir in swine influenza viruses, per N-subtype, the Netherlands, 2019–2023 (n = 36)

All swine influenza virus M2 amino acid sequences generated had one, two or three amino acid changes associated with driving resistance to M2-blockers (i.e. L26I, L26F, V27A, V27I, S31N). Among the PA sequences, two H1N1 viruses had amino acid substitution I38V that was previously reported to cause a two to fourfold reduction in susceptibility to baloxavir marboxil in human A(H1N1)pdm09 viruses. Determination of the phenotypic susceptibility to baloxavir of one of these two for which an isolate was available is pending. Looking at the NA sequences, seven N1 sequences had amino acid substitution Y155H, which causes highly reduced inhibition by oseltamivir and zanamivir in former seasonal human A(H1N1), but not in human A(H1N1)pdm09 viruses [28]. Sensitivity to oseltamivir and zanamivir was also tested phenotypically for 36 representative swine virus isolates (including two with NA-Y155H), providing evidence for sensitivity to these drugs that was in the normal range (no reduced inhibition) for all viruses tested (Figure 3). Of the highest half maximal inhibitory concentration (IC_50_) values, the fold-change compared with the median by N-subtype and neuraminidase inhibitor did not exceed the 10-fold threshold for reduced inhibition. Therefore, NA-Y155H found in the Dutch swine A(H1N1) viruses did not cause (highly) reduced inhibition, similar to human A(H1N1)pdm09 influenza viruses with NA-Y155H.

H: histidine; N: neuraminidase; Y: tyrosine; Y155H: viruses carrying NA-Y155H.

Black diamond dots indicate outliers after Tukey box and whisker plot analyses.

### Antigenic characterisation

The virus isolates for a genetically representative subset of viruses were characterised antigenically in the HI test using ferret antisera. Among the clade 1A.3.3.2 viruses, only A/Swine/Netherlands/719/19 cross-reacted with available antisera, e.g. those raised against H1N1pdm09 virus or clade 1C viruses. The new ferret antisera (A/Swine/Netherlands/719/19 and A/Swine/Netherlands/4322/20) recognised both a different subgroup of the clade 1A.3.3.2 viruses, but there was no cross-reactivity between these two subgroups (Figure 2). Additional antisera will be needed for better characterisation. None of the clade 1B.1.2.1 viruses reacted with the available antisera raised against clade 1A and 1C viruses, but reactions were measured with the 1B.1.2.1 sera and some antigenic diversity was observed also. Additional antisera will be needed for further characterisation. The clade 1C.2.1 and 1C.2.2 viruses reacted well with a set of antisera raised against older and contemporary 1C viruses, showing relatively modest antigenic diversity in this group. Tables with the raw HI data are shown in Supplementary Material.

## Discussion

The results described here confirm that swine influenza is enzootic on pig farms in the Netherlands and circulation appears to be year-round. On-farm virus circulation may be fuelled by the repeated influx of newborn young piglets on farrowing farms that are relatively naïve to the virus or arrival of naïve pigs on farms without an all-in/all-out system. In an all-in/all-out system, a batch of piglets enter, move and exit the production system together and the premises are emptied, cleaned and disinfected between the rounds. In the pilot, farms were predominantly positive (77/90; 85%) in their piglet population. A higher detection rate was found using group oral fluid samples, a result probably due to an increased probability of sampling pigs shedding the virus. As pig group (pen) size was not recorded, we cannot adjust for group size and compare differences in detection rates between sampling methods. From 342 influenza-positive samples, 125 (in)complete influenza A virus genomes were obtained: H1 viruses belonged to lineages 1A (clade 1A.3.3.2), 1B (clade 1B.1.2.1) and 1C (clades 1C.2.1 and 1C.2.2). Interestingly, HI testing of viruses from the HA 1A.3.3.2 clade identified a subgroup of viruses which were antigenically distinct from the other circulating viruses from this clade. These subgroups also cluster into two separate subclades within the 1A.3.3.2 clade. In line with these two clusters, a possible third introduction of human A(H1N1)pdm09 viruses may have resulted in yet another and potential antigenically distinct cluster within this 1A classical lineage. Unlike in other EU countries, no H1 HA clade 1C.2.4 and 1C.2.5 viruses were detected in our study, indicating that swine influenza in Europa occurs on a (sub)local scale [29]. Interestingly, only a single H3N2 virus was detected. This low H3N2 virus prevalence is in line with the observations from other European countries [1,29]. The closest virus sequence clustering with this virus was a German virus detected in 2016. Not much is known about the susceptibility of swine influenza viruses for antivirals. We showed that no reduced susceptibility for oseltamivir (mostly used in the Netherlands for therapy) and zanamivir was detected among the swIAV, reassuring options for treatment when a human gets infected and needs treatment. For baloxavir, which is not yet being used in the Netherlands, few viruses showed a marker for mild reduced susceptibility, but this has to be phenotypically confirmed in the context of swIAV.

The value of this survey became evident in August 2022, when a human infection with a H1N2 swIAV was detected in a patient involving contact with pigs, in human surveillance activities by study partners [7]. The human virus was genetically related to the recently collected swIAV in pigs. Based on the genome sequence, a well-founded interpretation of the antigenic properties could be made, which – due to the availability of reference viruses and antisera – could also be confirmed experimentally. More detailed clinical results for this and other recent swIAV infections in humans in the Netherlands can be found in the paper by Eggink et al. [7]. These occasional zoonotic and anthroponotic events demonstrate that swIAV concerns both swine and public health and stress the importance of monitoring (farm) animal populations, especially in countries like the Netherlands with high density of swine and human populations. Such programmes can be of benefit for the reduction of the disease burden in swine, for instance, as the antigenic characterisation of circulating viruses provide clues on the vaccine efficacy of current and future swine influenza vaccines. However, such programmes also help to identify emerging reassortant viruses that may be transmitted to humans, such as the recent H1pdm09N1av swine viruses found through passive swine surveillance in Denmark, for which little pre-existing immunity exits in humans [30].

## Conclusion

Overall, our findings provided insights into the circulating swIAVs in pigs in the Netherlands, the level of antiviral susceptibility, and the antigenic divergence between these viruses. It also showed that viruses from both humans and pigs occasionally switch host with possible implications for public health and veterinary health. The online molecular platform enabled sharing of valuable information of suspected zoonoses, monitoring of reassortment events and emergence of mutant viruses. It is an example of data sharing between domains at a national level. This project will be continued in the Netherlands and in collaboration with the European and Global influenza surveillance networks.

## Data Availability

All (sequence) data have been uploaded to the GISAID platform (https://gisaid.org).

## Supplementary Data

2914000 Supplementary Material
