# Supplementary material for: Swine influenza virus surveillance programme pilot to assess the risk for animal and public health, the Netherlands, 2022 to 2023
Source: Euro Surveill. 2025 Jun 5;30(22):2400664. doi: 10.2807/1560-7917.ES.2025.30.22.2400664 (PMC12143122; doi:10.2807/1560-7917.ES.2025.30.22.2400664)
Supplement: Supplementary Material [file 24-00664_VANDERVRIES_Supplement.pdf]

Swine influenza virus surveillance programme pilot to assess the risk for animal and public health, the Netherlands, 2022 to 2023

This supplementary material is hosted by Eurosurveillance as supporting information alongside the article Swine influenza virus surveillance programme pilot to assess the risk for animal and public health, the Netherlands, 2022 to 2023, on behalf of the authors, who remain responsible for the accuracy and appropriateness of the content. The same standards for ethics, copyright, attributions and permissions as for the article apply. Supplements are not edited by Eurosurveillance and the journal is not responsible for the maintenance of any links or email addresses provided therein.

In this Supplementary material, we display the corresponding influenza NA trees of H1 viruses and provide raw data of the HI assays.

Supplemental Figure 1. Phylogenetic tree for the NA of the detected H1N1 viruses. *Viruses isolated from pigs are coloured orange. Human reference viruses are coloured blue.*

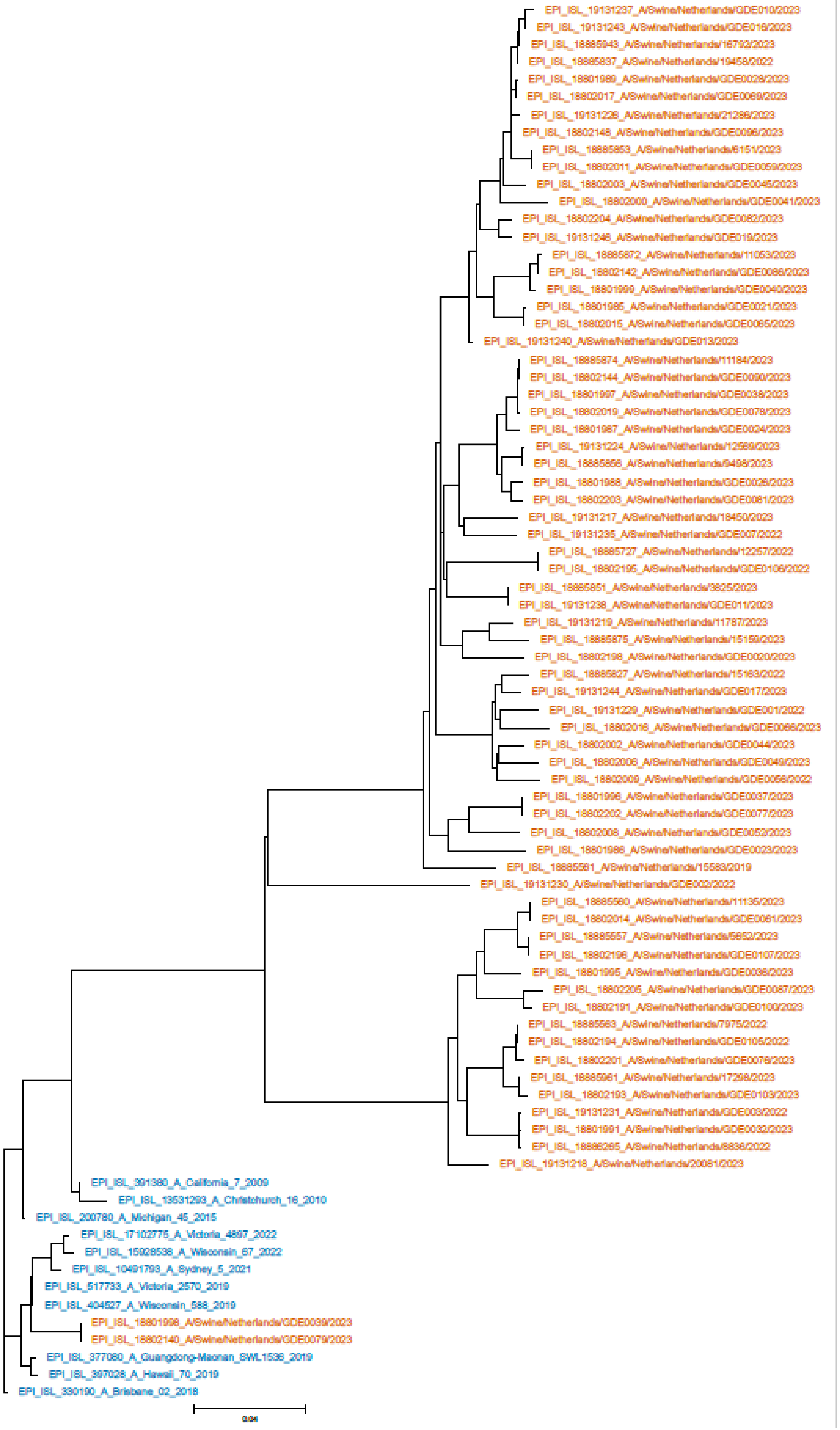
EPI_ISL_19131237_A/Swine/Netherlands/GDE010/2023
EPI_ISL_19131243_A/Swine/Netherlands/GDE016/2023
EPI_ISL_18885943_A/Swine/Netherlands/16792/2023
EPI_ISL_18885837_A/Swine/Netherlands/19458/2022
EPI_ISL_18801989_A/Swine/Netherlands/GDE0028/2023
EPI_ISL_18802017_A/Swine/Netherlands/GDE0069/2023
EPI_ISL_19131226_A/Swine/Netherlands/21286/2023
EPI_ISL_18802148_A/Swine/Netherlands/GDE0096/2023
EPI_ISL_18885853_A/Swine/Netherlands/6151/2023
EPI_ISL_18802011_A/Swine/Netherlands/GDE0059/2023
EPI_ISL_18802003_A/Swine/Netherlands/GDE0045/2023
EPI_ISL_18802000_A/Swine/Netherlands/GDE0041/2023
EPI_ISL_18802204_A/Swine/Netherlands/GDE0082/2023
EPI_ISL_19131246_A/Swine/Netherlands/GDE019/2023
EPI_ISL_18885872_A/Swine/Netherlands/11053/2023
EPI_ISL_18802142_A/Swine/Netherlands/GDE0086/2023
EPI_ISL_18801999_A/Swine/Netherlands/GDE0040/2023
EPI_ISL_18801985_A/Swine/Netherlands/GDE0021/2023
EPI_ISL_18802015_A/Swine/Netherlands/GDE0065/2023
EPI_ISL_19131240_A/Swine/Netherlands/GDE013/2023
EPI_ISL_18885874_A/Swine/Netherlands/11184/2023
EPI_ISL_18802144_A/Swine/Netherlands/GDE0090/2023
EPI_ISL_18801997_A/Swine/Netherlands/GDE0038/2023
EPI_ISL_18802019_A/Swine/Netherlands/GDE0078/2023
EPI_ISL_18801987_A/Swine/Netherlands/GDE0024/2023
EPI_ISL_19131224_A/Swine/Netherlands/12569/2023
EPI_ISL_18885856_A/Swine/Netherlands/9498/2023
EPI_ISL_18801988_A/Swine/Netherlands/GDE0026/2023
EPI_ISL_18802203_A/Swine/Netherlands/GDE0081/2023
EPI_ISL_19131217_A/Swine/Netherlands/18450/2023
EPI_ISL_19131235_A/Swine/Netherlands/GDE007/2022
EPI_ISL_18885727_A/Swine/Netherlands/12257/2022
EPI_ISL_18802195_A/Swine/Netherlands/GDE0106/2022
EPI_ISL_18885851_A/Swine/Netherlands/3825/2023
EPI_ISL_19131238_A/Swine/Netherlands/GDE011/2023
EPI_ISL_19131219_A/Swine/Netherlands/11787/2023
EPI_ISL_18885875_A/Swine/Netherlands/15159/2023
EPI_ISL_18802198_A/Swine/Netherlands/GDE0020/2023
EPI_ISL_18885827_A/Swine/Netherlands/15163/2022
EPI_ISL_19131244_A/Swine/Netherlands/GDE017/2023
EPI_ISL_19131229_A/Swine/Netherlands/GDE001/2022
EPI_ISL_18802016_A/Swine/Netherlands/GDE0066/2023
EPI_ISL_18802002_A/Swine/Netherlands/GDE0044/2023
EPI_ISL_18802006_A/Swine/Netherlands/GDE0049/2023
EPI_ISL_18802009_A/Swine/Netherlands/GDE0056/2022
EPI_ISL_18801996_A/Swine/Netherlands/GDE0037/2023
EPI_ISL_18802202_A/Swine/Netherlands/GDE0077/2023
EPI_ISL_18802008_A/Swine/Netherlands/GDE0052/2023
EPI_ISL_18801986_A/Swine/Netherlands/GDE0023/2023
EPI_ISL_18885561_A/Swine/Netherlands/15583/2019
EPI_ISL_19131230_A/Swine/Netherlands/GDE002/2022
EPI_ISL_18885560_A/Swine/Netherlands/11135/2023
EPI_ISL_18802014_A/Swine/Netherlands/GDE0061/2023
EPI_ISL_18885557_A/Swine/Netherlands/5852/2023
EPI_ISL_18802196_A/Swine/Netherlands/GDE0107/2023
EPI_ISL_18801995_A/Swine/Netherlands/GDE0036/2023
EPI_ISL_18802205_A/Swine/Netherlands/GDE0087/2023
EPI_ISL_18802191_A/Swine/Netherlands/GDE0100/2023
EPI_ISL_18885563_A/Swine/Netherlands/7975/2022
EPI_ISL_18802194_A/Swine/Netherlands/GDE0105/2022
EPI_ISL_18802201_A/Swine/Netherlands/GDE0076/2023
EPI_ISL_18885961_A/Swine/Netherlands/17298/2023
EPI_ISL_18802193_A/Swine/Netherlands/GDE0103/2023
EPI_ISL_19131231_A/Swine/Netherlands/GDE003/2022
EPI_ISL_18801991_A/Swine/Netherlands/GDE0032/2023
EPI_ISL_18886265_A/Swine/Netherlands/8836/2022
EPI_ISL_19131218_A/Swine/Netherlands/20081/2023
EPI_ISL_391380_A_California_7_2009
EPI_ISL_13531293_A_Christchurch_16_2010
EPI_ISL_200780_A_Michigan_45_2015
EPI_ISL_17102775_A_Victoria_4897_2022
EPI_ISL_15928538_A_Wisconsin_67_2022
EPI_ISL_10491793_A_Sydney_5_2021
EPI_ISL_517733_A_Victoria_2570_2019
EPI_ISL_404527_A_Wisconsin_588_2019
EPI_ISL_18801998_A/Swine/Netherlands/GDE0039/2023
EPI_ISL_18802140_A/Swine/Netherlands/GDE0079/2023
EPI_ISL_377080_A_Guangdong-Maonan_SWL1536_2019
EPI_ISL_397028_A_Hawaii_70_2019
EPI_ISL_330190_A_Brisbane_02_2018
0.04

Supplemental Figure 2. Phylogenetic tree for the NA of the detected H1N2 viruses.

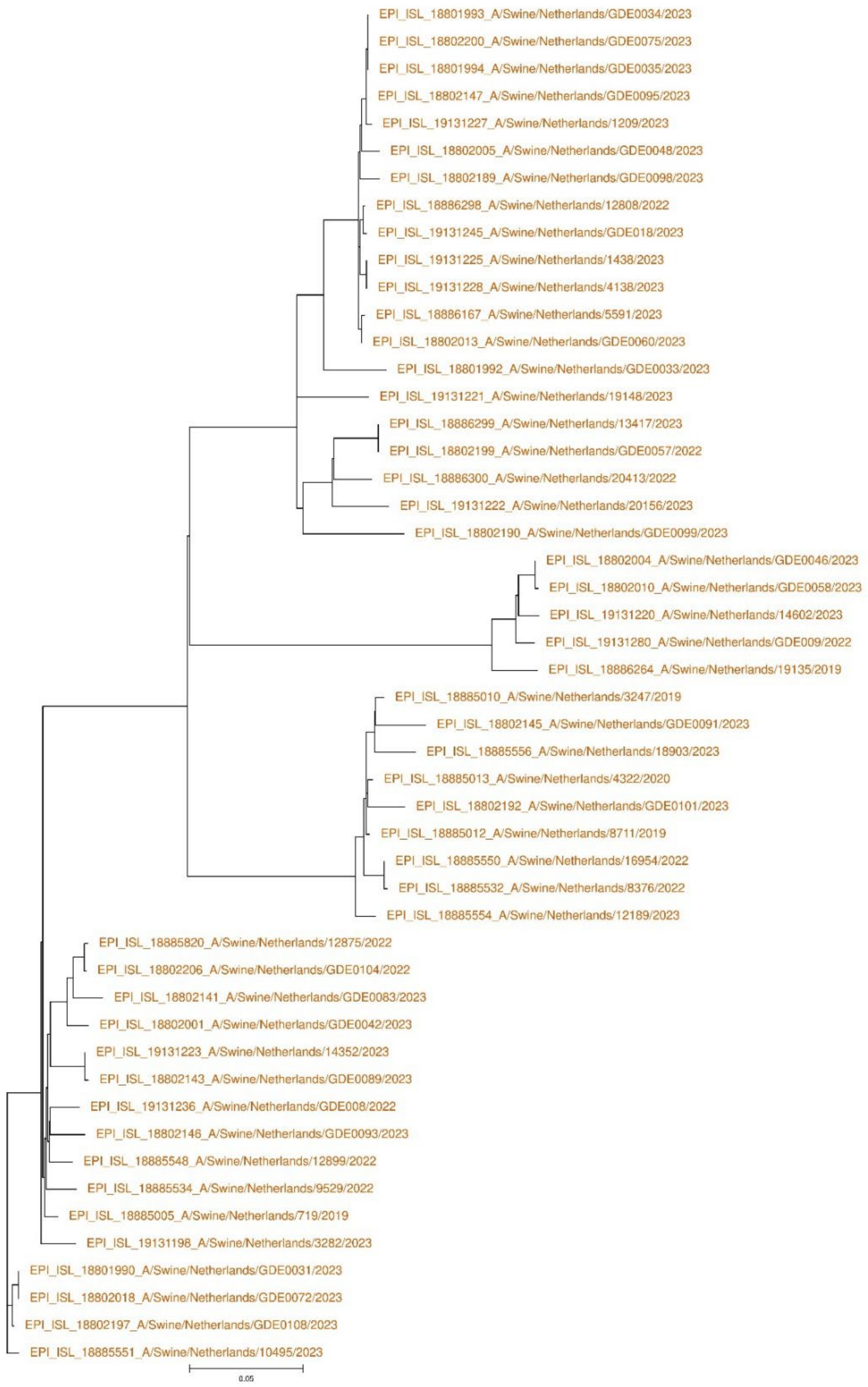

Supplemental Figure 3. *Radial H1 tree showing diversity of human (blue) and swine (orange) influenza viruses. Human viruses (n=193) were isolated from patients in the Netherlands between 2014-2023. Swine viruses (n=125) were from isolated from pigs on Dutch farms between 2022-2023.*

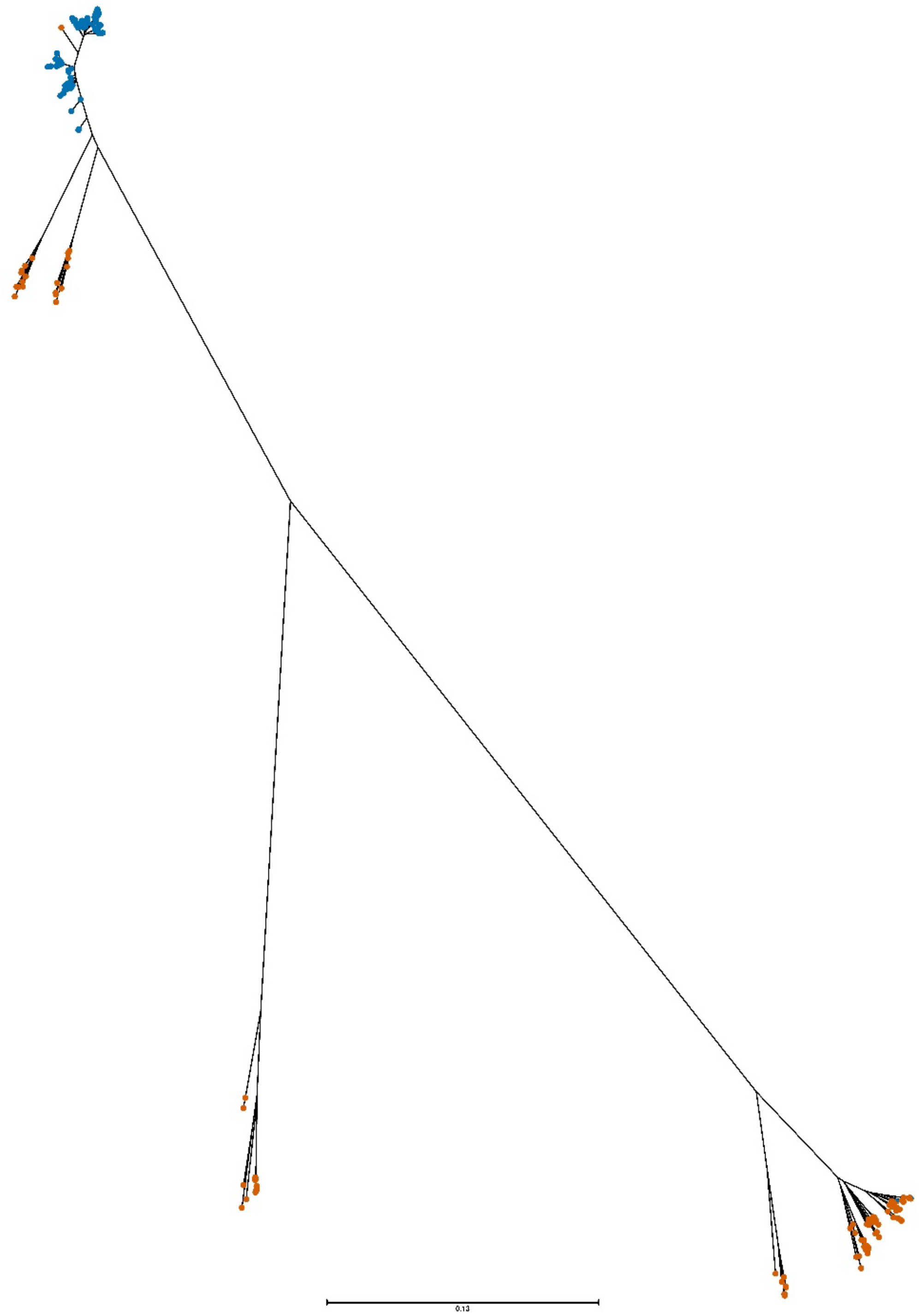

Supplemental figure 4. *HA tree showing the genetic diversity of H3 swine influenza viruses from Europe compared with those of human influenza viruses represented by human vaccine candidate strains since 2009. Human vaccine candidate strains are coloured blue. The recent swine H3N2 virus from the Netherlands is coloured red.*

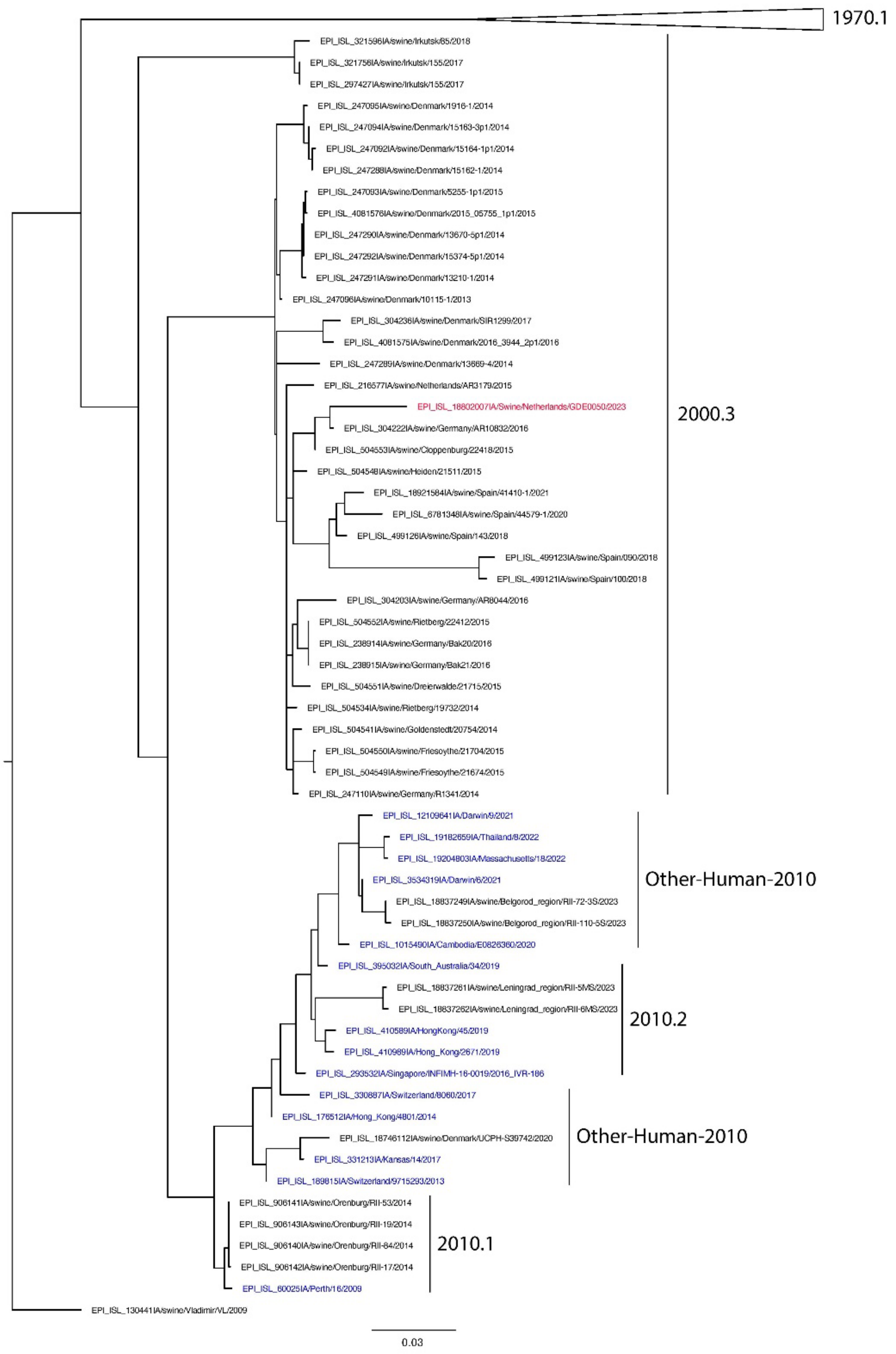
1970.1
EPI_ISL_321596IA/swine/Irkutsk/65/2018
EPI_ISL_321756IA/swine/Irkutsk/155/2017
EPI_ISL_297427IA/swine/Irkutsk/155/2017
EPI_ISL_247095IA/swine/Denmark/1916-1/2014
EPI_ISL_247094IA/swine/Denmark/15163-3p1/2014
EPI_ISL_247092IA/swine/Denmark/15164-1p1/2014
EPI_ISL_247288IA/swine/Denmark/15162-1/2014
EPI_ISL_247093IA/swine/Denmark/5255-1p1/2015
EPI_ISL_4081576IA/swine/Denmark/2015_05755_1p1/2015
EPI_ISL_247290IA/swine/Denmark/13670-5p1/2014
EPI_ISL_247292IA/swine/Denmark/15374-5p1/2014
EPI_ISL_247291IA/swine/Denmark/13210-1/2014
EPI_ISL_247096IA/swine/Denmark/10115-1/2013
EPI_ISL_304236IA/swine/Denmark/SIR1299/2017
EPI_ISL_4081575IA/swine/Denmark/2016_3944_2p1/2016
EPI_ISL_247289IA/swine/Denmark/13669-4/2014
EPI_ISL_216577IA/swine/Netherlands/AR3179/2015
EPI_ISL_18802007IA/Swine/Netherlands/GDE0050/2023
EPI_ISL_304222IA/swine/Germany/AR10832/2016
EPI_ISL_504553IA/swine/Cloppenburg/22418/2015
EPI_ISL_504548IA/swine/Heiden/21511/2015
EPI_ISL_18921584IA/swine/Spain/41410-1/2021
EPI_ISL_6781348IA/swine/Spain/44579-1/2020
EPI_ISL_499126IA/swine/Spain/143/2018
EPI_ISL_499123IA/swine/Spain/090/2018
EPI_ISL_499121IA/swine/Spain/100/2018
EPI_ISL_304203IA/swine/Germany/AR8044/2016
EPI_ISL_504552IA/swine/Rietberg/22412/2015
EPI_ISL_238914IA/swine/Germany/Bak20/2016
EPI_ISL_238915IA/swine/Germany/Bak21/2016
EPI_ISL_504551IA/swine/Dreierwalde/21715/2015
EPI_ISL_504534IA/swine/Rietberg/19732/2014
EPI_ISL_504541IA/swine/Goldenstedt/20754/2014
EPI_ISL_504550IA/swine/Friesoythe/21704/2015
EPI_ISL_504549IA/swine/Friesoythe/21674/2015
EPI_ISL_247110IA/swine/Germany/R1341/2014
2000.3
EPI_ISL_12109641IA/Darwin/9/2021
EPI_ISL_19182659IA/Thailand/8/2022
EPI_ISL_19204803IA/Massachusetts/18/2022
EPI_ISL_3534319IA/Darwin/6/2021
EPI_ISL_18837249IA/swine/Belgorod_region/RII-72-3S/2023
EPI_ISL_18837250IA/swine/Belgorod_region/RII-110-5S/2023
EPI_ISL_1015490IA/Cambodia/E0826360/2020
Other-Human-2010
EPI_ISL_395032IA/South_Australia/34/2019
EPI_ISL_18837261IA/swine/Leningrad_region/RII-5MS/2023
EPI_ISL_18837262IA/swine/Leningrad_region/RII-6MS/2023
EPI_ISL_410589IA/HongKong/45/2019
EPI_ISL_410989IA/Hong_Kong/2671/2019
EPI_ISL_293532IA/Singapore/INFIMH-16-0019/2016_IVR-186
2010.2
EPI_ISL_330887IA/Switzerland/8060/2017
EPI_ISL_176512IA/Hong_Kong/4801/2014
EPI_ISL_18746112IA/swine/Denmark/UCPH-S39742/2020
EPI_ISL_331213IA/Kansas/14/2017
EPI_ISL_189815IA/Switzerland/9715293/2013
Other-Human-2010
EPI_ISL_906141IA/swine/Orenburg/RII-53/2014
EPI_ISL_906143IA/swine/Orenburg/RII-19/2014
EPI_ISL_906140IA/swine/Orenburg/RII-84/2014
EPI_ISL_906142IA/swine/Orenburg/RII-17/2014
EPI_ISL_60025IA/Perth/16/2009
2010.1
EPI_ISL_130441IA/swine/Vladimir/VL/2009
0.03

Supplemental Table 1. *Virus isolates, accession numbers, HA and NA subtypes and HA clade assignment*

Virus	GISAID ID	HA Subtype	NA Subtype	HA Clade
Influenza A/Swine/Netherlands/GDE001/2022	EPI_ISL_19131229	H1	N1	1C.2.2
Influenza A/Swine/Netherlands/GDE002/2022	EPI_ISL_19131230	H1[*]	N1	N/A
Influenza A/Swine/Netherlands/GDE003/2022	EPI_ISL_19131231	H1[*]	N1	N/A
Influenza A/Swine/Netherlands/GDE004/2022	EPI_ISL_19131232	H1[*]	N1	N/A
Influenza A/Swine/Netherlands/GDE005/2022	EPI_ISL_19131233	H1[*]	N2	N/A
Influenza A/Swine/Netherlands/GDE006/2022	EPI_ISL_19131234	H1[*]	N1	N/A
Influenza A/Swine/Netherlands/GDE007/2022	EPI_ISL_19131235	H1	N1	1C.2.2
Influenza A/Swine/Netherlands/GDE008/2022	EPI_ISL_19131236	H1	N2	1A.3.3.2
Influenza A/Swine/Netherlands/GDE009/2022	EPI_ISL_19131280	H1	N2	1C.2.2
Influenza A/Swine/Netherlands/GDE010/2023	EPI_ISL_19131237	H1[*]	N1	N/A
Influenza A/Swine/Netherlands/GDE011/2023	EPI_ISL_19131238	H1[*]	N1	N/A
Influenza A/Swine/Netherlands/GDE012/2023	EPI_ISL_19131239	H1	N1	1C.2.2
Influenza A/Swine/Netherlands/GDE013/2023	EPI_ISL_19131240	H1	N1	N/A
Influenza A/Swine/Netherlands/GDE014/2023	EPI_ISL_19131241	H1	N1	1C.2.2
Influenza A/Swine/Netherlands/GDE015/2023	EPI_ISL_19131242	H1	N1	1A.3.3.2
Influenza A/Swine/Netherlands/GDE016/2023	EPI_ISL_19131243	H1	N1	1C.2.2
Influenza A/Swine/Netherlands/GDE017/2023	EPI_ISL_19131244	H1	N1	1C.2.2
Influenza A/Swine/Netherlands/GDE018/2023	EPI_ISL_19131245	H1	N2	1B.1.2.1
Influenza A/Swine/Netherlands/GDE019/2023	EPI_ISL_19131246	H1	N1	1C.2.2
Influenza A/Swine/Netherlands/10495/2023	EPI_ISL_18885551	H1	N2	1A.3.3.2
Influenza A/Swine/Netherlands/11135/2023	EPI_ISL_18885560	H1	N1	1C.2.1
Influenza A/Swine/Netherlands/12257/2022	EPI_ISL_18885727	H1	N1	1C.2.2
Influenza A/Swine/Netherlands/12808/2022	EPI_ISL_18886298	H1	N2	1B.1.2.1
Influenza A/Swine/Netherlands/12875/2022	EPI_ISL_18885820	H1	N2	1C.2.2
Influenza A/Swine/Netherlands/12899/2022	EPI_ISL_18885548	H1	N2	1A.3.3.2
Influenza A/Swine/Netherlands/13417/2023	EPI_ISL_18886299	H1	N2	1B.1.2.1
Influenza A/Swine/Netherlands/15163/2022	EPI_ISL_18885827	H1	N1	1C.2.2
Influenza A/Swine/Netherlands/15583/2019	EPI_ISL_18885561	H1	N1	1C.2.2
Influenza A/Swine/Netherlands/16954/2022	EPI_ISL_18885550	H1	N2	1A.3.3.2
Influenza A/Swine/Netherlands/18903/2023	EPI_ISL_18885556	H1	N2	1A.3.3.2
Influenza A/Swine/Netherlands/19135/2019	EPI_ISL_18886264	H1	N2	1C.2.2
Influenza A/Swine/Netherlands/19458/2022	EPI_ISL_18885837	H1	N1	1C.2.2
Influenza A/Swine/Netherlands/20413/2022	EPI_ISL_18886300	H1	N2	1B.1.2.1
Influenza A/Swine/Netherlands/3247/2019	EPI_ISL_18885010	H1	N2	1A.3.3.2
Influenza A/Swine/Netherlands/3282/2023	EPI_ISL_19131198	H1	N2	1A.3.3.2
Influenza A/Swine/Netherlands/3825/2023	EPI_ISL_18885851	H1	N1	1C.2.2
Influenza A/Swine/Netherlands/4322/2020	EPI_ISL_18885013	H1	N2	1A.3.3.2
Influenza A/Swine/Netherlands/5591/2023	EPI_ISL_18886167	H1	N2	1B.1.2.1
Influenza A/Swine/Netherlands/5652/2023	EPI_ISL_18885557	H1	N1	1C.2.1
Influenza A/Swine/Netherlands/6151/2023	EPI_ISL_18885853	H1	N1	1C.2.2
Influenza A/Swine/Netherlands/719/2019	EPI_ISL_18885005	H1	N2	1A.3.3.2

Influenza A/Swine/Netherlands/7975/2022	EPI_ISL_18885563	H1	N1	1C.2.2
Influenza A/Swine/Netherlands/8376/2022	EPI_ISL_18885532	H1	N2	1A.3.3.2
Influenza A/Swine/Netherlands/8836/2022	EPI_ISL_18886265	H1	N1	1B.1.2.1
Influenza A/Swine/Netherlands/9529/2022	EPI_ISL_18885534	H1	N2	1A.3.3.2
Influenza A/Swine/Netherlands/8711/2019	EPI_ISL_18885012	H1	N2	1A.3.3.2
Influenza A/Swine/Netherlands/GDE0021/2023	EPI_ISL_18801985	H1	N1	1C.2.2
Influenza A/Swine/Netherlands/GDE0023/2023	EPI_ISL_18801986	H1	N1	1C.2.2
Influenza A/Swine/Netherlands/GDE0024/2023	EPI_ISL_18801987	H1	N1	1C.2.2
Influenza A/Swine/Netherlands/GDE0026/2023	EPI_ISL_18801988	H1	N1	1C.2.2
Influenza A/Swine/Netherlands/GDE0028/2023	EPI_ISL_18801989	H1	N1	1C.2.2
Influenza A/Swine/Netherlands/GDE0031/2023	EPI_ISL_18801990	H1	N2	1A.3.3.2
Influenza A/Swine/Netherlands/GDE0032/2023	EPI_ISL_18801991	H1	N1	1B.1.2.1
Influenza A/Swine/Netherlands/GDE0033/2023	EPI_ISL_18801992	H1	N2	1C.2.2
Influenza A/Swine/Netherlands/GDE0034/2023	EPI_ISL_18801993	H1	N2	1B.1.2.1
Influenza A/Swine/Netherlands/GDE0035/2023	EPI_ISL_18801994	H1	N2	1B.1.2.1
Influenza A/Swine/Netherlands/GDE0036/2023	EPI_ISL_18801995	H1	N1	1C.2.1
Influenza A/Swine/Netherlands/GDE0037/2023	EPI_ISL_18801996	H1	N1	1C.2.2
Influenza A/Swine/Netherlands/GDE0038/2023	EPI_ISL_18801997	H1	N1	1C.2.2
Influenza A/Swine/Netherlands/GDE0039/2023	EPI_ISL_18801998	H1	N1	1A.3.3.2
Influenza A/Swine/Netherlands/GDE0040/2023	EPI_ISL_18801999	H1	N1	1C.2.2
Influenza A/Swine/Netherlands/GDE0041/2023	EPI_ISL_18802000	H1	N1	1C.2.2
Influenza A/Swine/Netherlands/GDE0042/2023	EPI_ISL_18802001	H1	N2	1C.2.2
Influenza A/Swine/Netherlands/GDE0044/2023	EPI_ISL_18802002	H1	N1	1C.2.2
Influenza A/Swine/Netherlands/GDE0045/2023	EPI_ISL_18802003	H1	N1	1C.2.2
Influenza A/Swine/Netherlands/GDE0046/2023	EPI_ISL_18802004	H1	N2	1C.2.2
Influenza A/Swine/Netherlands/GDE0048/2023	EPI_ISL_18802005	H1	N2	1A.3.3.2
Influenza A/Swine/Netherlands/GDE0049/2023	EPI_ISL_18802006	H1	N1	1C.2.2
Influenza A/Swine/Netherlands/GDE0050/2023	EPI_ISL_18802007	H3	N2	H3
Influenza A/Swine/Netherlands/GDE0052/2023	EPI_ISL_18802008	H1	N1	1C.2.2
Influenza A/Swine/Netherlands/GDE0056/2022	EPI_ISL_18802009	H1	N1	1C.2.2
Influenza A/Swine/Netherlands/GDE0058/2023	EPI_ISL_18802010	H1	N2	1C.2.2
Influenza A/Swine/Netherlands/GDE0059/2023	EPI_ISL_18802011	H1	N1	1C.2.2
Influenza A/Swine/Netherlands/GDE0060/2023	EPI_ISL_18802013	H1	N2	1B.1.2.1
Influenza A/Swine/Netherlands/GDE0061/2023	EPI_ISL_18802014	H1	N1	1C.2.1
Influenza A/Swine/Netherlands/GDE0065/2023	EPI_ISL_18802015	H1	N1	1C.2.2
Influenza A/Swine/Netherlands/GDE0066/2023	EPI_ISL_18802016	H1	N1	1C.2.2
Influenza A/Swine/Netherlands/GDE0069/2023	EPI_ISL_18802017	H1	N1	1C.2.2
Influenza A/Swine/Netherlands/GDE0072/2023	EPI_ISL_18802018	H1	N2	1A.3.3.2
Influenza A/Swine/Netherlands/GDE0078/2023	EPI_ISL_18802019	H1	N1	1C.2.2
Influenza A/Swine/Netherlands/GDE0079/2023	EPI_ISL_18802140	H1	N1	1A.3.3.2
Influenza A/Swine/Netherlands/GDE0083/2023	EPI_ISL_18802141	H1	N2	1C.2.2
Influenza A/Swine/Netherlands/GDE0086/2023	EPI_ISL_18802142	H1	N1	1C.2.2
Influenza A/Swine/Netherlands/GDE0089/2023	EPI_ISL_18802143	H1	N2	1A.3.3.2
Influenza A/Swine/Netherlands/GDE0090/2023	EPI_ISL_18802144	H1	N1	1C.2.2
Influenza A/Swine/Netherlands/GDE0091/2023	EPI_ISL_18802145	H1	N2	1A.3.3.2

Influenza A/Swine/Netherlands/GDE0093/2023	EPI_ISL_18802146	H1	N2	1A.3.3.2
Influenza A/Swine/Netherlands/GDE0095/2023	EPI_ISL_18802147	H1	N2	1B.1.2.1
Influenza A/Swine/Netherlands/GDE0096/2023	EPI_ISL_18802148	H1	N1	1C.2.2
Influenza A/Swine/Netherlands/GDE0098/2023	EPI_ISL_18802189	H1	N2	1B.1.2.1
Influenza A/Swine/Netherlands/GDE0099/2023	EPI_ISL_18802190	H1	N2	1B.1.2.1
Influenza A/Swine/Netherlands/GDE0100/2023	EPI_ISL_18802191	H1	N1	1C.2.1
Influenza A/Swine/Netherlands/GDE0101/2023	EPI_ISL_18802192	H1	N2	1A.3.3.2
Influenza A/Swine/Netherlands/GDE0103/2023	EPI_ISL_18802193	H1	N1	1C.2.2
Influenza A/Swine/Netherlands/GDE0105/2022	EPI_ISL_18802194	H1	N1	1C.2.2
Influenza A/Swine/Netherlands/GDE0106/2022	EPI_ISL_18802195	H1	N1	1C.2.2
Influenza A/Swine/Netherlands/GDE0107/2023	EPI_ISL_18802196	H1	N1	1C.2.1
Influenza A/Swine/Netherlands/GDE0108/2023	EPI_ISL_18802197	H1	N2	1A.3.3.2
Influenza A/Swine/Netherlands/GDE0020/2023	EPI_ISL_18802198	H1	N1	1C.2.2
Influenza A/Swine/Netherlands/GDE0057/2022	EPI_ISL_18802199	H1	N2	1B.1.2.1
Influenza A/Swine/Netherlands/GDE0075/2023	EPI_ISL_18802200	H1	N2	1B.1.2.1
Influenza A/Swine/Netherlands/GDE0076/2023	EPI_ISL_18802201	H1	N1	1C.2.2
Influenza A/Swine/Netherlands/GDE0077/2023	EPI_ISL_18802202	H1	N1	1C.2.2
Influenza A/Swine/Netherlands/GDE0081/2023	EPI_ISL_18802203	H1	N1	1C.2.2
Influenza A/Swine/Netherlands/GDE0082/2023	EPI_ISL_18802204	H1	N1	1C.2.2
Influenza A/Swine/Netherlands/GDE0087/2023	EPI_ISL_18802205	H1	N1	1C.2.1
Influenza A/Swine/Netherlands/GDE0104/2022	EPI_ISL_18802206	H1	N2	1C.2.2
Influenza A/Swine/Netherlands/20081/2023	EPI_ISL_19131218	H1	N1	1C.2.2
Influenza A/Swine/Netherlands/11787/2023	EPI_ISL_19131219	H1	N1	1C.2.2
Influenza A/Swine/Netherlands/11053/2023	EPI_ISL_18885872	H1	N1	1C.2.2
Influenza A/Swine/Netherlands/14602/2023	EPI_ISL_19131220	H1	N2	1C.2.1
Influenza A/Swine/Netherlands/19148/2023	EPI_ISL_19131221	H1	N2	1A.3.3.2
Influenza A/Swine/Netherlands/20156/2023	EPI_ISL_19131222	H1	N2	1C.2.1
Influenza A/Swine/Netherlands/14352/2023	EPI_ISL_19131223	H1	N2	1A.3.3.2
Influenza A/Swine/Netherlands/11184/2023	EPI_ISL_18885874	H1	N1	1C.2.2
Influenza A/Swine/Netherlands/9498/2023	EPI_ISL_18885856	H1	N1	1C.2.2
Influenza A/Swine/Netherlands/12569/2023	EPI_ISL_19131224	H1	N1	1C.2.2
Influenza A/Swine/Netherlands/1438/2023	EPI_ISL_19131225	H1	N2	1B.1.2.1
Influenza A/Swine/Netherlands/21286/2023	EPI_ISL_19131226	H1	N1	1C.2.2
Influenza A/Swine/Netherlands/17298/2023	EPI_ISL_18885961	H1	N1	1C.2.2
Influenza A/Swine/Netherlands/16792/2023	EPI_ISL_18885943	H1	N1	1C.2.2
Influenza A/Swine/Netherlands/1209/2023	EPI_ISL_19131227	H1	N2	1B.1.2.1
Influenza A/Swine/Netherlands/12189/2023	EPI_ISL_18885554	H1	N2	1A.3.3.2
Influenza A/Swine/Netherlands/4138/2023	EPI_ISL_19131228	H1	N2	1B.1.2.1
Influenza A/Swine/Netherlands/18450/2023	EPI_ISL_19131217	H1	N1	1C.2.2
Influenza A/Swine/Netherlands/15159/2023	EPI_ISL_18885875	H1	N1	1C.2.2
**As determined by subtype specific PCR*				

Supplemental Table 2. *Antigenic characterization of "classical" swine influenza viruses from the Netherlands by HI assay with turkey erythrocytes and ferret antisera. Homologous titers are shown in bold and underlined. Viruses and sera for clade 1A.3.3.2 generated in this study are shown in the gray-shaded area.*

			Hebei/1572/19	NL/3315/16	NL/10370-1b/20	Sw/NL/11135/22	Hessen/47/20	Sw/NL/6151/23	Cal/7/09	Sw/NL/719/19	Sw/NL/4322/20
Hebei-haigang/1572/19	H1N1v	1C.2.3	**<u>2560</u>**	240	960	480	2560	960	2560	1920	<10
NL/3315/16	H1N1v	1C.2.1	640	**<u>2560</u>**	320	480	40	480	<10	<10	<10
NL/10370-1b/20	H1N1v	1C.2.1	1920	320	**<u>1280</u>**	640	960	640	960	320	<10
Sw/NL/11135/22	H1N1	1C.2.1	1920	240	960	**<u>1280</u>**	960	480	2560	480	<10
Hessen/47/20	H1N1v	1C.2.2	2560	<10	320	120	**<u>3840</u>**	480	960	960	<10
Sw/NL/6151/23	H1N1	1C.2.2	640	40	480	240	640	**<u>1920</u>**	1280	320	<10
Cal/7/09	H1N1pdm		1920	<10	960	480	1920	960	**<u>7680</u>**	2560	<10
Sw/NL/719/19	H1N2	1A.3.3.2	320	<10	160	80	640	240	960	**<u>2560</u>**	<10
Sw/NL/9529/22	H1N2	1A.3.3.2	<10	<10	<10	<10	<10	<10	80	2560	<10
Sw/NL/12899/22	H1N2	1A.3.3.2	<10	<10	<10	10	<10	<10	80	2560	<10
Sw/NL/10495/22	H1N2	1A.3.3.2	<10	<10	<10	<10	<10	<10	160	1280	<10
Sw/NL/4322/20	H1N2	1A.3.3.2	<10	<10	<10	<10	<10	<10	<10	<10	**<u>640</u>**
Sw/NL/8711/19	H1N2	1A.3.3.2	<10	<10	<10	<10	<10	<10	<10	<10	320
Sw/NL/12189/22	H1N2	1A.3.3.2	<10	<10	<10	<10	<10	<10	<10	<10	160
Sw/NL/18903/22	H1N2	1A.3.3.2	<10	<10	<10	<10	<10	<10	<10	<10	160
Sw/NL/3247/19	H1N2	1A.3.3.2	<10	<10	<10	<10	<10	<10	<10	<10	2560
Sw/NL/16954/22	H1N2	1A.3.3.2	<10	<10	<10	<10	<10	<10	<10	<10	2560
Sw/NL/8376/22	H1N2	1A.3.3.2	<10	<10	<10	<10	<10	<10	<10	<10	2560

Supplemental Table 3. *Antigenic characterization of "human seasonal" swine influenza viruses from the Netherlands by HI assay with turkey erythrocytes and ferret antisera. Homologous titers are shown in bold and underlined. Viruses and sera for clade 1B.1.2.1 generated in this study are shown in the gray-shaded area.*

			Cal/7/09	Sw/NL/719/19	Sw/NL/4322/20	Hebei/1572/19	NL/3315/16	NL/10370-1b/20	Sw/NL/11135/22	Hessen/47/20	Sw/NL/6151/23	Sw/NL/8836/22	Sw/NL/5591/22
Cal/7/09	H1N1pdm		**7680**	2560	<10	1920	<10	960	480	1920	960	<10	<10
Sw/NL/719/19	H1N2	1A.3.3.2	960	**2560**	<10	320	<10	160	80	640	240	<10	<10
Sw/NL/4322/20	H1N2	1A.3.3.2	<10	<10	**640**	<10	<10	<10	<10	<10	<10	<10	<10
Hebei-haigang/1572/19	H1N1v	1C.2.3	2560	1920	<10	**2560**	240	960	480	2560	960	<10	<10
NL/3315/16	H1N1v	1C.2.1	<10	<10	<10	640	**2560**	320	480	40	480	<10	<10
NL/10370-1b/20	H1N1v	1C.2.1	960	320	<10	1920	320	**1280**	640	960	640	<10	<10
Sw/NL/11135/22	H1N1	1C.2.1	2560	480	<10	1920	240	960	**1280**	960	480	40	<10
Hessen/47/20	H1N1v	1C.2.2	960	960	<10	2560	<10	320	120	**3840**	480	<10	<10
Sw/NL/6151/23	H1N1	1C.2.2	1280	320	<10	640	40	480	240	640	**1920**	<10	<10
Sw/NL/8836/22	H1N1	1B.1.2.1	<10	<10	<10	<10	<10	<10	<10	<10	<10	**3840**	320
Sw/NL/13417/22	H1N2	1B.1.2.1	<10	<10	<10	<10	<10	<10	<10	<10	<10	640	960
Sw/NL/5591/22	H1N2	1B.1.2.1	160	<10	<10	<10	<10	<10	<10	<10	<10	160	**2560**
Sw/NL/1209/22	H1N2	1B.1.2.1	<10	<10	<10	<10	<10	<10	<10	<10	<10	480	2560
Sw/NL/12808/22	H1N2	1B.1.2.1	<10	<10	<10	<10	<10	<10	<10	<10	<10	320	2560
Sw/NL/20413/22	H1N2	1B.1.2.1	20	<10	<10	<10	<10	<10	<10	<10	<10	320	2560

Supplemental Table 4. *Antigenic characterization of “Eurasian avian” swine influenza viruses from the Netherlands by HI assay with turkey erythrocytes and ferret antisera. Homologous titers are shown in bold and underlined. Viruses and sera for clade 1C.2.1 and 1C.2.2 generated in this study are shown in the gray-shaded area.*

			Sw/NL/5591/22	Sw/NL/8836/22	Sw/NL/4322/20	Cal/7/09	Sw/NL/719/19	NL/386/86	Sw/Best/5M/96	Hebei-haigang/1572/19	NL/3315/16	NL/10370-1b/20	Sw/NL/11135/22	Hessen/47/20	Sw/NL/6151/23
Sw/NL/5591/22	H1N2	1B.1.2.1	**2560**	160	<10	160	<10	NT	NT	<10	<10	<10	<10	<10	<10
Sw/NL/8836/22	H1N1	1B.1.2.1	320	**3840**	<10	<10	<10	NT	NT	<10	<10	<10	<10	<10	<10
Sw/NL/4322/20	H1N2	1A.3.3.2	<10	<10	**640**	<10	<10	NT	NT	<10	<10	<10	<10	<10	<10
Cal/7/09	H1N1pdm		<10	<10	<10	**7680**	2560	80	<10	1920	<10	960	480	1920	960
Sw/NL/719/19	H1N2	1A.3.3.2	<10	<10	<10	960	**2560**	NT	NT	320	<10	160	80	640	240
NL/386/1986	H1N1v	1C	<10	<10	<10	1920	960	**480**	240	1280	30	960	320	1280	1280
Sw/Best/5M/96	H1N1	1C	NT	NT	NT	NT	NT	1280	**1920**	320	240	40	NT	10	NT
Hebei-haigang/1572/19	H1N1v	1C.2.3	<10	<10	<10	2560	1920	240	<10	**2560**	180	640	480	1920	960
NL/3315/16	H1N1v	1C.2.1	<10	<10	<10	<10	<10	NT	NT	640	**2560**	320	480	40	480
NL/10370-1b/20	H1N1v	1C.2.1	<10	<10	<10	960	320	80	<10	1280	240	**1280**	640	640	640
Sw/NL/11135/22	H1N1	1C.2.1	<10	40	<10	2560	480	160	<10	1280	200	640	**1280**	640	480
Hessen/47/20	H1N1v	1C.2.2	<10	<10	<10	960	960	80	<10	1920	<10	240	120	**2560**	480
Sw/NL/6151/23	H1N1	1C.2.2	<10	<10	<10	1280	320	80	<10	480	40	320	240	480	**1920**
Sw/NL/5652/22	H1N1	1C.2.1	<10	<10	<10	<10	<10	40	<10	320	80	160	640	80	320
Sw/NL/12875/22	H1N2	1C.2.2	<10	<10	<10	1280	320	40	<10	320	<10	160	320	320	1280
Sw/NL/19458/22	H1N1	1C.2.2	<10	<10	<10	1920	640	80	<10	320	40	120	320	320	1280
Sw/NL/15583/19	H1N1	1C.2.2	<10	<10	<10	1920	1280	80	<10	960	80	160	320	640	1280
Sw/NL/15163/22	H1N1	1C.2.2	<10	<10	<10	1280	1280	120	<10	640	160	80	320	640	1280
Sw/NL/3825/22	H1N1	1C.2.2	<10	<10	<10	1920	1280	160	20	640	240	240	240	640	2560
Sw/NL/7975/22	H1N1	1C.2.2	<10	<10	<10	1920	480	160	<10	480	40	320	320	480	640
Sw/NL/19135/19	H1N2	1C.2.2	<10	<10	<10	2560	2560	240	<10	1280	80	480	320	1280	1280
Sw/NL/12257/22	H1N1	1C.2.2	<10	<10	<10	2560	1920	320	<10	1280	40	480	320	1280	1280
Sw/NL/11053/22	H1N1	1C.2.2	<10	<10	<10	640	320	NT	NT	640	640	1280	640	640	1280
Sw/NL/16792/22	H1N1	1C.2.2	<10	<10	<10	960	320	NT	NT	640	80	640	320	640	960
Sw/NL/17298/22	H1N1	1C.2.2	<10	<10	<10	1920	320	NT	NT	960	10	640	240	960	640
Sw/NL/9498/22	H1N1	1C.2.2	<10	<10	<10	640	640	NT	NT	1920	20	320	240	2560	640
Sw/NL/11184/22	H1N1	1C.2.2	<10	<10	<10	1280	1280	NT	NT	2560	40	640	320	2560	1280
Sw/NL/15159/22	H1N1	1C.2.2	<10	<10	<10	1280	2560	NT	NT	2560	120	1280	960	2560	1920
Sw/NL/20121/22	H1N1	1C.2.2	<10	<10	<10	480	240	NT	NT	640	80	640	320	640	640
Sw/NL/15331/22	H1N1	1C.2.2	<10	<10	<10	1280	1280	NT	NT	2560	120	1280	960	2560	1920
